# Locomotor adaptability in persons with unilateral transtibial amputation

**DOI:** 10.1371/journal.pone.0181120

**Published:** 2017-07-12

**Authors:** Benjamin J. Darter, Amy J. Bastian, Erik J. Wolf, Elizabeth M. Husson, Bethany A. Labrecque, Brad D. Hendershot

**Affiliations:** 1 Department of Physical Therapy, Virginia Commonwealth University, Richmond, Virginia, United States of America; 2 Department of Research, Hunter Holmes McGuire Veteran Affairs Medical Center, Richmond, Virginia, United States of America; 3 Kennedy Krieger Institute, Baltimore, Maryland, United States of America; 4 Department of Neuroscience, Johns Hopkins University School of Medicine, Baltimore, Maryland, United States of America; 5 Department of Rehabilitation, Walter Reed National Military Medical Center, Bethesda, Maryland, United States of America; 6 DoD-VA Extremity Trauma and Amputation Center of Excellence (EACE), Bethesda, Maryland, United States of America; 7 BADER Consortium, University of Delaware, Newark, Delaware, United States of America; 8 Department of Rehabilitation Medicine, Uniformed Services University of the Health Sciences, Bethesda, Maryland, United States of America; University of Colorado Boulder, UNITED STATES

## Abstract

**Background:**

Locomotor adaptation enables walkers to modify strategies when faced with challenging walking conditions. While a variety of neurological injuries can impair locomotor adaptability, the effect of a lower extremity amputation on adaptability is poorly understood.

**Objective:**

Determine if locomotor adaptability is impaired in persons with unilateral transtibial amputation (TTA).

**Methods:**

The locomotor adaptability of 10 persons with a TTA and 8 persons without an amputation was tested while walking on a split-belt treadmill with the parallel belts running at the same (tied) or different (split) speeds. In the split condition, participants walked for 15 minutes with the respective belts moving at 0.5 m/s and 1.5 m/s. Temporal spatial symmetry measures were used to evaluate reactive accommodations to the perturbation, and the adaptive/de-adaptive response.

**Results:**

Persons with TTA and the reference group of persons without amputation both demonstrated highly symmetric walking at baseline. During the split adaptation and tied post-adaptation walking both groups responded with the expected reactive accommodations. Likewise, adaptive and de-adaptive responses were observed. The magnitude and rate of change in the adaptive and de-adaptive responses were similar for persons with TTA and those without an amputation. Furthermore, adaptability was no different based on belt assignment for the prosthetic limb during split adaptation walking.

**Conclusions:**

Reactive changes and locomotor adaptation in response to a challenging and novel walking condition were similar in persons with TTA to those without an amputation. Results suggest persons with TTA have the capacity to modify locomotor strategies to meet the demands of most walking conditions despite challenges imposed by an amputation and use of a prosthetic limb.

## Introduction

Restoring a rudimentary walking ability in persons with a lower extremity amputation is a fundamental goal of physical rehabilitation. In the majority of instances, especially among younger individuals, this goal can be met[1]. However, no device yet fully replicates the motor or sensory functions of the amputated structures[2]. Walking performance is therefore altered, and the occurrence of undesirable outcomes is increased (e.g., biomechanical deviations, increased physiological energy cost for walking, increased risk for falling)[3–6]. These undesirable outcomes may become even more prominent and troublesome when faced with challenging walking conditions.

In walking, it is generally accepted that locomotor patterns are stored in spinal cord neurons (central pattern generators- CPG)[7]. Afferent feedback to the spinal cord provides some reactive flexibility in the locomotor patterns to achieve step-to-step postural balance and stability. Whereas feedback to supraspinal structures like the cerebellum enables a predictive feedforward trial-and-error process to adapt existing movement patterns or potentially acquire new ones[7–9]. Interactions between reactive feedback and predictive feedforward control systems underlie the locomotor adaptability used to meet the demands of a wide variety of potentially challenging walking conditions[10].

Recent research examining locomotor adaptability has used a split-belt treadmill walking paradigm (unequal treadmill belt speeds) to create a perturbation known to result in well-defined and reliable changes in temporal-spatial measures[11]. First, the unequal belt speeds cause an immediate reactive asymmetry in many temporal-spatial characteristics, including step length, stance time and limb excursion (or stride length as named in other studies[11–13]). Second, this asymmetry decreases as the individual continues to walk (despite unequal belt speeds), reflecting predictive feedforward adaptation to the locomotor strategy. Improved step length symmetry offers the clearest evidence of an adaptive response as longer step lengths by the limb on the slow belt and shorter step lengths by the limb on the fast belt gradually become more equal. Third, an aftereffect of the unequal speeds occurs when both belts return to a common speed. This aftereffect appears as longer step lengths for the limb assigned to the fast belt during the split-belt walking than the limb previously assigned to the slow belt. The asymmetry is replaced by a more symmetrical pattern as the aftereffect washes out with continued walking. The improved symmetry reflects de-adaptation or switching away from the locomotor strategy utilized during the split-belt condition. “Adaptation” and “de-adaptation” may be observed in other parameters including stance time symmetry and limb excursion symmetry, but the responses are less common and may simply be changes necessary to avoid instability during split-belt treadmill testing[13].

Results from a variety of populations, including stroke survivors, and persons with traumatic brain injury or Parkinson’s disease, reveal impaired locomotor adaptability can occur when the normal flow of information within the nervous system is altered[12, 14, 15]. Specifically, those with impaired adaptability are more perturbed (demonstrate greater asymmetry) and/or adapt/de-adapt more slowly[16, 17]. The potential for impairment also exists when sensory information from the periphery is altered, such as would occur when an extremity is amputated. The loss of motor control provided through a prosthetic limb may only compound the risk for impaired adaptability. Although literature describing the effect of an amputation on locomotor adaptability is limited, recent studies provide some insight on reactive and adaptive changes when walking is perturbed using a split-belt treadmill[18, 19]. Specifically, one study suggested persons with transtibial amputation (TTA) and those without amputation were equally perturbed at the onset of the unequal walking speeds based on similar magnitude reactive accommodations in step length symmetry[18]. Furthermore, the results indicated persons with TTA relied on a center of mass (CoM) displacement strategy when adapting step lengths. Interestingly, the group with TTA adopted the CoM displacement strategy earlier than those without an amputation, potentially because of a reliance on the intact limb to compensate for an inability to increase prosthetic ankle work. However, statistical testing was not included comparing the reactive changes or the rate of adaptation for those with and without amputation. Of note, the prosthetic side was always assigned to the faster moving treadmill belt; it is therefore unclear if adaptive changes would differ if the prosthetic side were assigned to the slow belt.

The purpose of this study was to assess the effects of a lower extremity amputation on locomotor adaptability using a split-belt treadmill paradigm. We hypothesized participants with amputation would respond to the locomotor perturbation with similar reactive increases in asymmetry for step length, limb excursion, and stance time as participants without an amputation. We also hypothesized the improvement in step length symmetry resulting from predictive feedforward adaptation/de-adaptation would occur at a faster rate in participants with amputation than those without amputation, and would be dependent on belt assignment for the prosthetic limb.

## Methods

### Participants

A convenience sample of persons with traumatic unilateral TTA as well as a reference group of healthy adults without amputation were recruited to participate in the study. Participants could have no known neurologic or orthopedic condition that impaired walking ability, excluding the presence of an amputation. An ability to walk continuously for 15 minutes without an assistive device (e.g., cane or walker) was required. Furthermore, individuals with prior experience walking on a split-belt treadmill with the belts moving at unequal speeds were excluded from the study. Participants with amputation were required to complete testing wearing their customary prosthetic device. All study participants provided written informed consent prior to completing the testing procedures approved by the Walter Reed National Military Medical Center Institutional Review Board.

### Testing protocol

**Fig 1:**
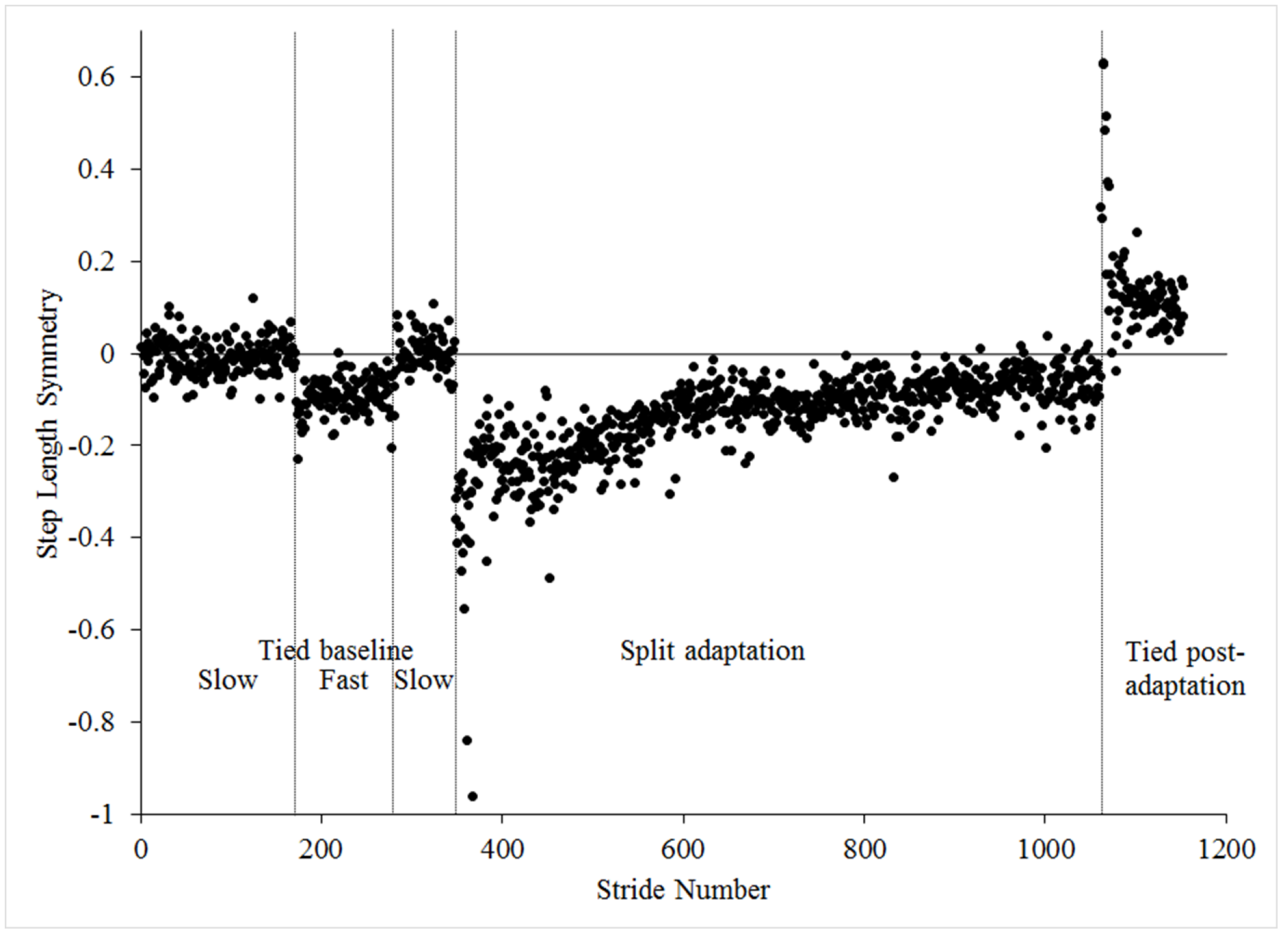
Example data from a single person with TTA. Tied baseline included walking with both treadmill belts at a slow speed (0.5 m/s) before and after a tied condition at a fast speed (1.5 m/s). During split adaptation the treadmill belts moved at either the slow or fast speed (3:1 speed ratio). Tied post-adaptation walking with both treadmill belts moving at the slow speed.

Testing was completed on a split-belt treadmill (Bertec Corporation, Columbus, OH). The following walking conditions were created by running the side-by-side belts at equal (“tied”) or different speeds (“split”) according to a well-established testing procedure: 1) tied acclimation, 2) tied baseline, 3) split adaptation, and 4) tied post-adaptation[11]. First, participants walked at a self-selected pace for four minutes to acclimate to the treadmill. Step length data collected during the last 30 seconds determined belt assignments for the split walking condition. A common characteristic of persons with TTA is step length asymmetry and during testing the leg taking a shorter step length was assigned to the fast belt during split-belt walking[20, 21]. This belt assignment strategy was selected because improved step length symmetry is a short-term aftereffect of split-belt walking in those who exhibit baseline asymmetries[14]. Belt assignment for the persons without an amputation was randomized as an inclusion criterion required a mean step length asymmetry < 0.04 m. Following treadmill acclimation, participants completed two baseline conditions: 1) five minutes with tied slow (0.5 m/s) belts and 2) two minutes with tied fast (1.5 m/s) belts. Participants were then exposed to a brief ten-second familiarization trial of split adaptation walking in which one belt was abruptly accelerated to three times faster (1.5 m/s) than the slow belt (0.5 m/s). This trial was included to minimize the potential for an exaggerated response when first exposed to split adaptation walking[22]. Participants then returned to a slow tied belt condition for two minutes to wash out any potential effects of the familiarization trial. Participants next completed a full fifteen minutes of split adaptation walking. Testing concluded with a tied post-adaptation condition consisting of both belts at the tied slow speed for five minutes. Fig 1 provides an example of the response in step length symmetry for a single participant completing the tested walking conditions.

Participants wore a safety harness during the testing that prevented falls but did not provide body weight support. Participants were permitted to use the right and left side treadmill handrails as needed, but guided to minimize weight-bearing through the rails and stop handrail use as soon as comfortable to do so. Furthermore, participants were asked to refrain from looking at the belts and instead asked to focus on programing of interest on a television monitor placed directly in front of the treadmill. The use of visual feedback, as well as a conscious attention on the adaptive process are known to affect the adaptive process[23–25]. Watching the monitor served to minimize these potential confounding effects.

### Data collection

Full-body kinematic data were collected at 120 Hz using a six-degrees of freedom marker set and a 27-camera motion capture system (Vicon, Oxford, UK)[26]. Analog data from force plates integrated into the treadmill were synchronously collected at 1200 Hz. Force plate data were used to identify initial contact (heel strike) and toe-off gait events in Visual 3D motion analysis software (C-Motion, Germantown, MD). All data were collected in 30 second trials throughout each walking condition.

### Data reduction

Reactive accommodation and predictive feedforward changes during split-belt testing are routinely measured using a symmetry ratio for selected temporal-spatial parameters. Step length, limb excursion, and stance time symmetry were calculated as measures of the reactive changes. Step length symmetry was also the primary measurement of interest for predictive feedforward changes due to its robust adaptive response[11]. The symmetry ratio was defined as the difference between the lower extremity assigned to the fast belt during the split condition (L_fast_), and the limb assigned to the slow belt (L_slow_):

$$\begin{array}{cc} \text{Symmetry }= & \left({\text{L}}_{\text{fast}}–{\text{L}}_{\text{slow}}\right)\\ & \left({\text{L}}_{\text{fast}}+{\text{L}}_{\text{slow}}\right) \end{array}$$ (1)

Step length (SL) was defined as the anterior—posterior distance between contralateral heel strikes for a mid-malleolus point representing the foot segment. Limb excursion was the anterior—posterior distance between heel strike and subsequent toe off. Stance time was the time between a limb’s heel strike and toe off. A value of 0 equated to “perfect” symmetry. Positive or negative values indicated the degree to which locomotion was perturbed. Furthermore, negative values indicated the spatial distance or time for the limb on the slow belt were longer than the limb on the fast belt. Positive values reflected the inverse. Normalization was performed to enable comparisons of participants with different sized temporal-spatial characteristics.

Reactive accommodation and predictive feedforward adaptation and de-adaptation were quantified using the magnitude and rate of change in symmetry. The magnitude, reflecting the degree of locomotion perturbation, was defined using averages of the last 5 strides of the baseline tied conditions (fast and slow), as well as the first and last 5 strides of the split adaptation (split-early and split-late) and tied post-adaptation (post-early and post-late) conditions. Rates of adaptation and de-adaption were determined by changes in step length symmetry (in 5 stride epochs) over the first 50 strides of the split adaptation and tied post-adaptation walking conditions[27].

### Statistical analysis

Two-sample t-tests were used to compare demographic characteristics between groups. Individual two-factor (group and walking condition) repeated measures mixed model analysis of variance (ANOVA) with follow-up post-hoc Tukey adjusted t-tests were used to evaluate differences in symmetry for the selected temporal spatial parameters. Moreover, ANOVA models were used to compare rates of the adaptive/de-adaptive response between the participants with TTA and those who did not have an amputation, as well as a comparison of participants with TTA based on treadmill belt assignment for the prosthetic limb. All hypothesis testing was evaluated at the significance level 0.05 using SAS 9.4.

## Results

Demographic information (Table 1) for the 10 persons with a unilateral traumatic TTA (mean ± SD: 32.2 ± 6.9 yr, 1.79 ± 0.06 m, 90.1 ± 14.2 kg) and 8 persons without an amputation (27.5 ± 6.9 yr, 1.79 ± 0.05 m, 86.3 ± 13.3 kg) were statistically similar (all p>.17). The participants with TTA used their customary prosthetic limb consisting of a suction socket, with in most cases a sleeve suspension, and equivalent dynamic energy storing and return feet. Step length measurements for persons with TTA during treadmill acclimation resulted in equal numbers of participants with the prosthetic limb assigned to the slow (n = 5) and fast belts (n = 5) during the split adaptation condition. All participants completed the testing protocol without difficulty. However, prolonged handrail use was observed more frequently in persons with TTA during split adaptation walking. Kinematic data for the upper extremities indicated 4 persons without amputation released the handrails within the first 5 seconds of split adaptation walking, 3 others released the handrails by 80 second, and only 1 held on the entire time. Whereas, 1 person with TTA released the handrails with 5 seconds, 3 released by 80 seconds in, and 3 held on the whole time. Handrails were also used during tied post-adaptation walking. However, unlike the split adaptation condition, use was similar with 7 of 8 persons without amputation, and 8 of 10 persons with TTA releasing the handrails within the first 30 seconds.

**Table 1:** Demographics for participants with transtibial amputation.

	Gender	Age (yrs)	Height (m)	Weight (kg)	Time Since Amputation (months)	Socket Type	Suspension Type	Prosthetic Foot	Prosthetic Belt Assignment
**1**	M	38	1.74	97.4	6	Carbon Fiber	Suction with sleeve	Variflex XC	Slow
**2**	M	39	1.72	93.1	5	Thermolyn	Suction with sleeve	Soleus Tactical	Slow
**3**	M	34	1.78	95.3	8	Thermolyn	Suction with sleeve	Variflex XC	Slow
**4**	M	23	1.73	70.9	4	Thermolyn	Suction with sleeve	Variflex XC	Fast
**5**	M	28	1.88	73.1	3	Thermolyn	Suction with sleeve	Variflex XC	Fast
**6**	M	25	1.76	77.2	3	Thermolyn	Elevated vacuum	Re-flex Rotate	Slow
**7**	M	35	1.83	83.6	12	Carbon Fiber	Suction with sleeve	Soleus Tactical	Slow
**8**	M	23	1.79	100.2	9	Carbon Fiber	Suction with sleeve	Kinterra	Fast
**9**	M	42	1.89	117.5	6	Carbon Fiber	Suction with sleeve	Variflex XC	Fast
**10**	M	35	1.805	92.8	63	Carbon Fiber	Pin-lock	Soleus Tactical	Fast
**Mean**		32.2	1.79	90.1	11.9				
**SD**		6.9	0.06	14.2	18.1				

SD: Standard Deviation

### Reactive feedback accommodation

#### Magnitude of reactive accommodation

**Fig 2:**
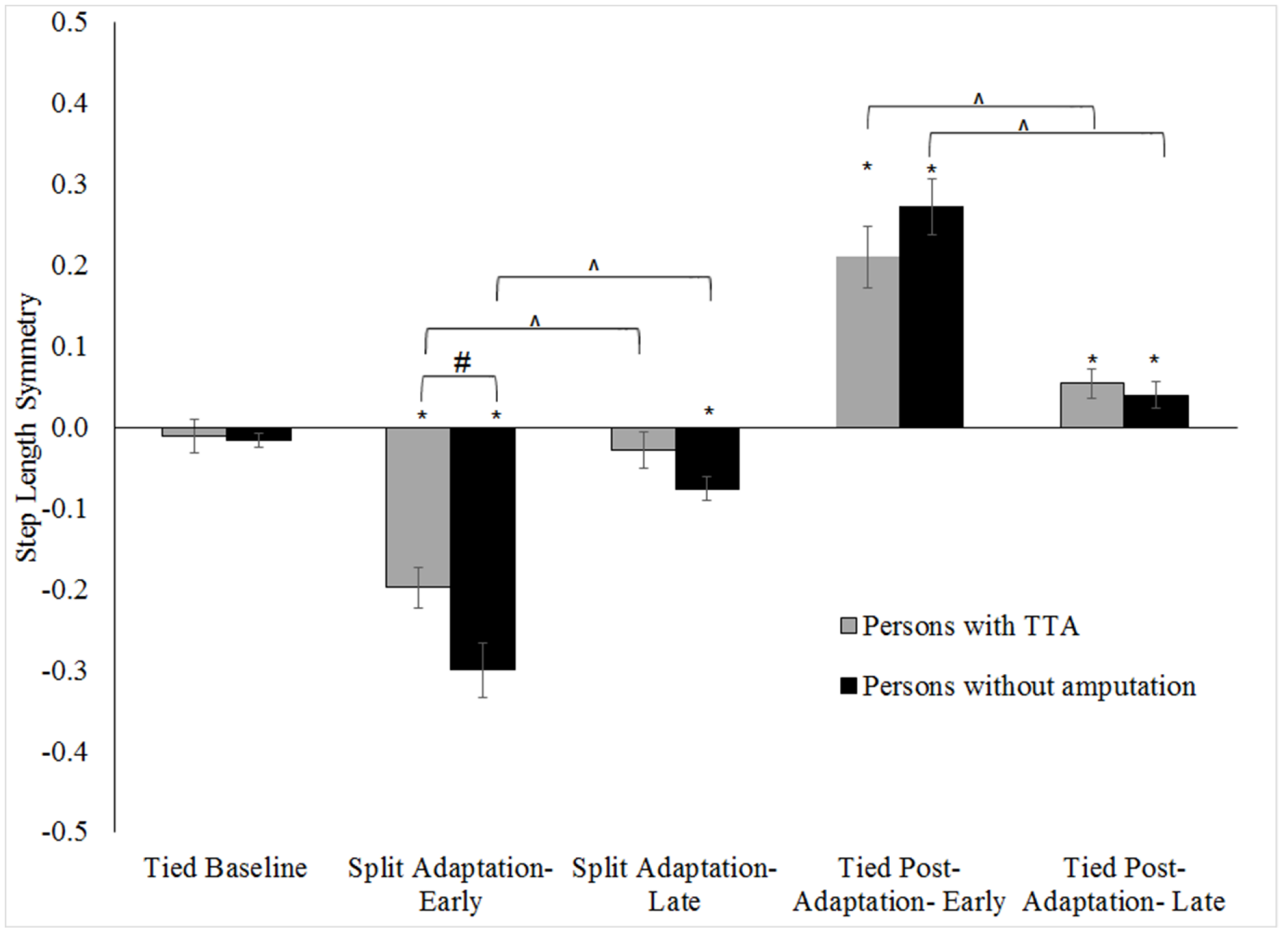
Comparison of step length symmetry across walking conditions for the persons with TTA and persons without an amputation. Error bars indicate standard error. * Indicates within-group differences comparing conditions to the tied baseline (0.5 m/s) were statistically significance with p <.05. # Indicates the between group difference for a walking condition was statistically significance with p <.05. ^ indicates a statistically significant change during split adaptation or tied post-adaptation with p <.05. Note: Increased handrail use by persons with TTA resulted in more symmetrical step lengths than the persons without an amputation during early split adaptation.

**Fig 3:**
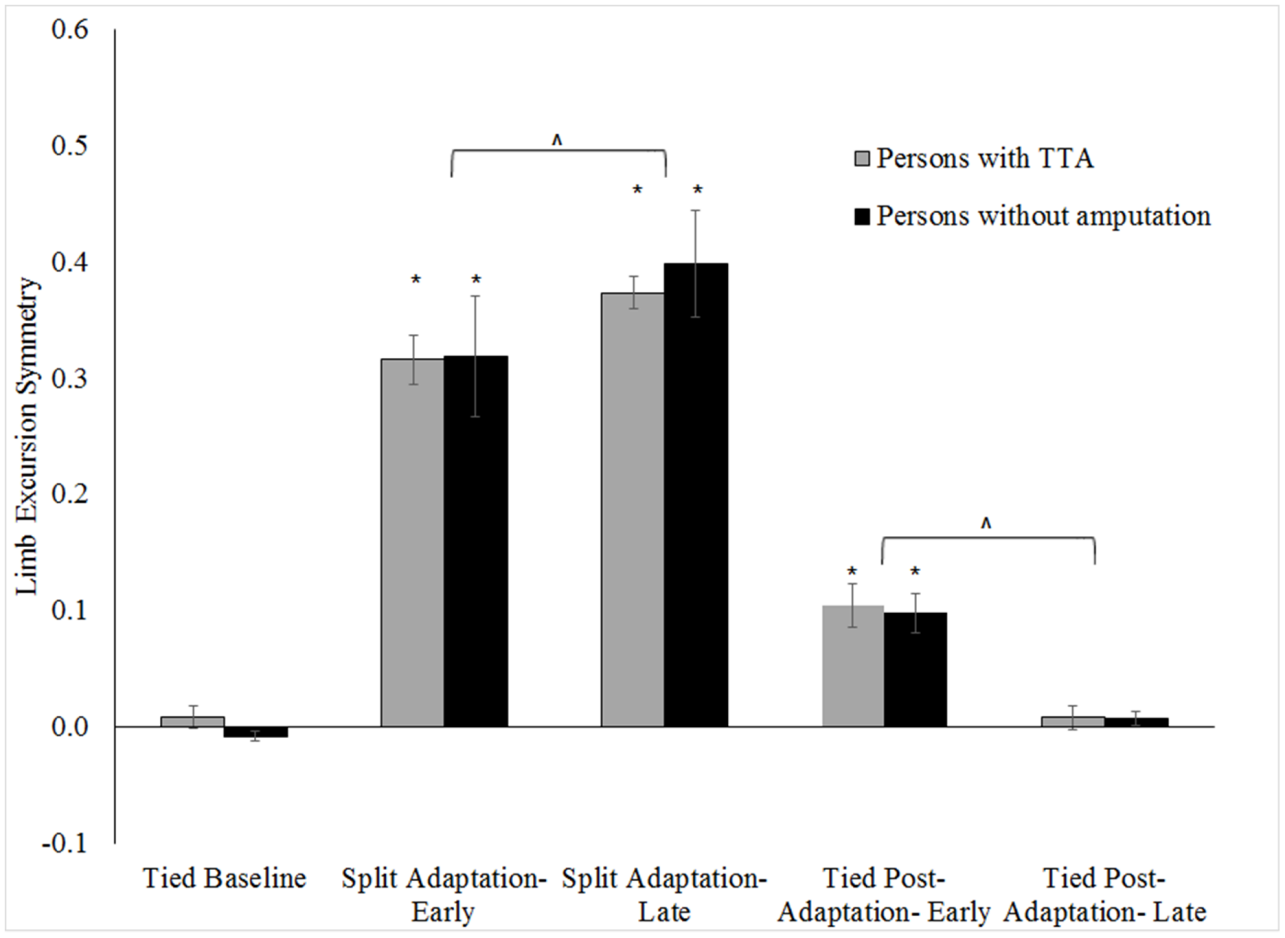
Comparison of limb excursion symmetry across walking conditions for the persons with TTA and persons without an amputation. Error bars indicate standard error. * Indicates within-group differences comparing conditions to the tied baseline (0.5 m/s) were statistically significant with p <.05. ^ indicates a statistically significant change during split adaptation or tied post-adaptation with p <.05.

**Fig 4:**
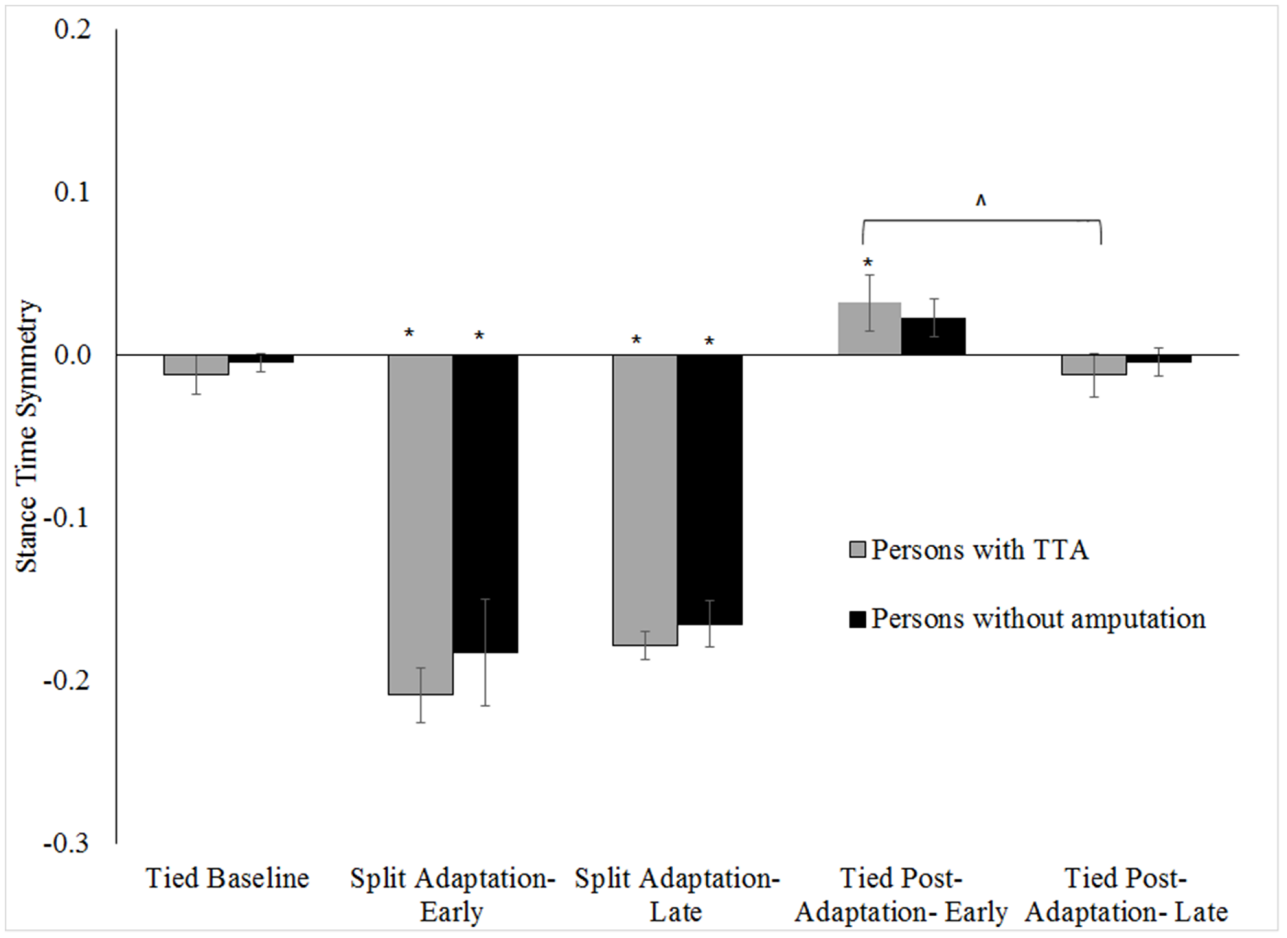
Comparison of stance time symmetry across walking conditions for the persons with TTA and persons without an amputation. Error bars indicate standard error. * Indicates within-group differences comparing conditions to the tied baseline (0.5 m/s) were statistically significant with p <.05. ^ indicates a statistically significant changes during tied post-adaptation with p <.05.

Within-group and between-group differences in step length, limb excursion and stance time symmetry for the tested walking conditions are shown in Figs 2–4 respectively (note, only slow baseline is presented as there was no difference in symmetry values between the slow and fast walking speeds; range of all p = .16-.78). Step length, limb excursion and stance time were highly symmetric during the tied baseline walking regardless of group. Whereas, large step length (all p <.01), limb excursion (all p <.01) and stance time (all p <.01) asymmetries were exhibited by each group at the start of split adaptation. The asymmetry resulted from longer step lengths and stance times for the limb on the slow belt relative to the limb on the fast belt, and longer excursions for the limb on the fast belt relative to the limb on the slow belt. Stance time symmetry was no different at the start of tied post-adaptation walking in the persons without an amputation (p = .58) but was for persons with TTA (p <.01). Conversely, step length and limb excursion symmetries differed from baseline values during early tied post-adaptation (all p <.01) for all participants. Step lengths became longer for the limb assigned to the fast belt during the split adaptation condition. This was opposite to the interlimb relationship of the split adaptation condition. The results reflect the known adaptive/de-adaptive response for step length symmetry detailed in the following section describing predictive feedforward based changes.

**Fig 5:**
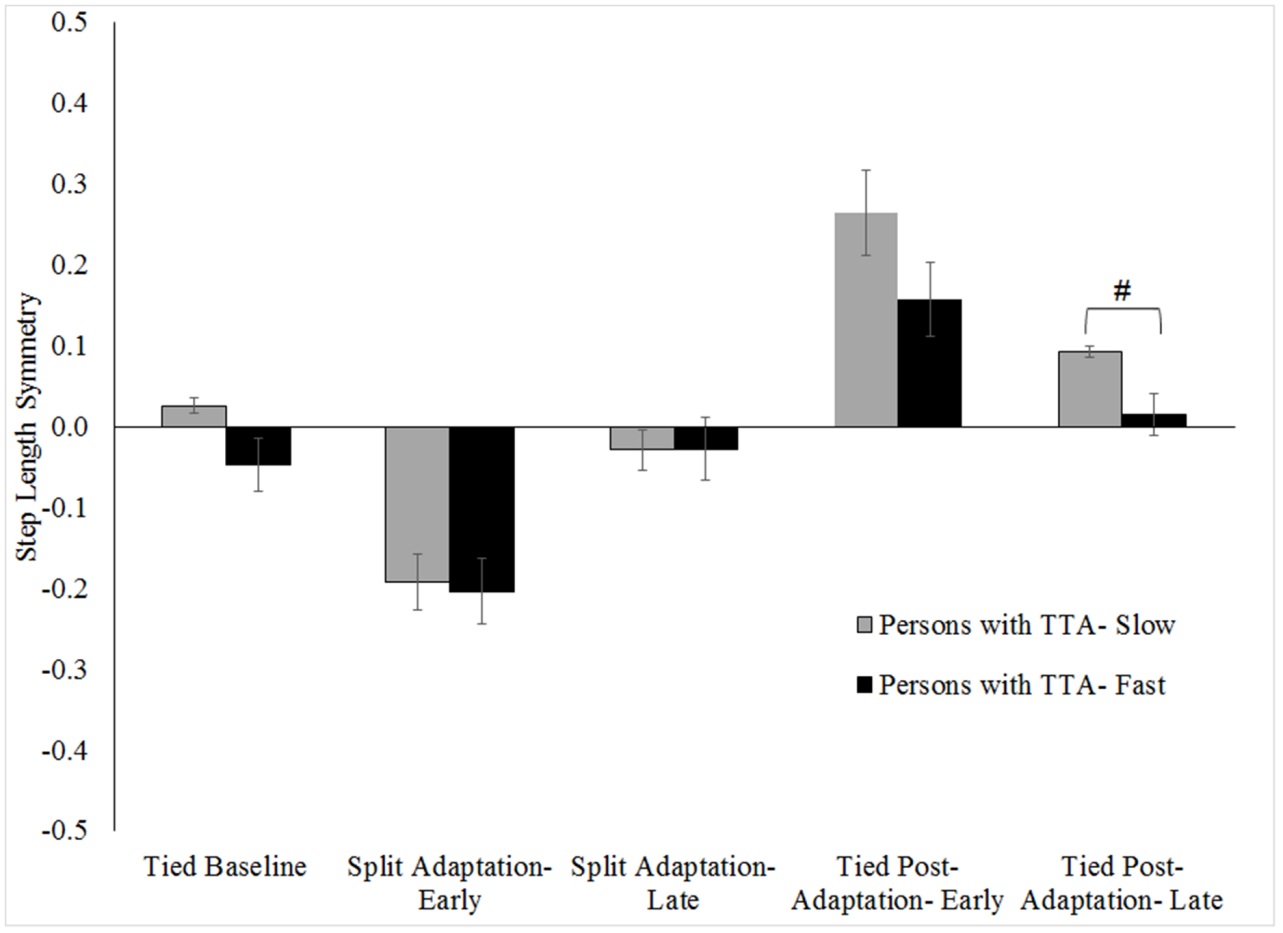
Comparison of step length symmetry across walking conditions for persons with TTA grouped by belt assignment for the prosthetic limb. Error bars indicate standard error. # Indicates the between group difference for a walking condition was statistically significant with p <.05.

No group level differences (persons with TTA vs. persons without an amputation) in any symmetry measure were found during baseline walking (all p>.15). Likewise, limb excursion and stance time symmetries were similar between groups for split adaptation and tied post-adaptation walking (all p>.45). However, results indicated persons with TTA were less perturbed during early split adaptation (walked more symmetrically) than the persons without an amputation (p = .02). No difference in step length symmetry was found among the persons with TTA based on belt assignment (Fig 5) during baseline (p = 0.06), split adaptation (p = 0.83) or tied post-adaptation walking (p = 0.16).

### Predictive feedforward adaptation

#### Magnitude of adaptation

Limb excursion changed in both groups during the split adaptation (persons with TTA p = .01; persons without amputation p <.01) and tied post-adaptation (all p <.01) conditions (Fig 3). Significant asymmetry in limb excursion remained at the end of split adaptation compared to baseline (all p <.01), and symmetry equivalent to baseline was found at the conclusion of tied post-adaptation walking in the persons with TTA (p = .96) but not the persons with amputation (p = .04).

Stance time symmetry did not change in either group during split adaptation (all p>.16; Fig 4) and remained statistically different than baseline at the end of split adaptation walking (all p>.01). However, stance time symmetry did improve in persons with TTA during tied post-adaptation walking (p <.01), but not in persons without an amputation (p = .11). Nevertheless, stance time symmetry was equivalent to baseline at the end of tied post-adaptation walking in both groups (all p>.97).

The step length asymmetry provoked by split adaptation walking improved over the course of the split adaptation and tied post-adaptation conditions in both groups of participants (all p <.01; Fig 2). Relative to baseline, step length symmetry was statistically equivalent at the end of the split adaptation walking for the persons with TTA (p = .48) but not for the persons without an amputation (p <.01). By the conclusion of tied post-adaptation both groups remained more asymmetric than baseline (p <.01). However, post-hoc t-tests on the absolute magnitude of asymmetry (p = .07 for the group without amputation; p = .20 for the group with amputation) showed the statistically significant finding resulted from a switch in which limb took a longer step and not because the asymmetry was more pronounced. Group level comparisons revealed no differences in the magnitude of the adaptation in any symmetry measure during split adaptation or tied post-adaptation walking (all p>.16).

#### Rate of adaptation

**Fig 6:**
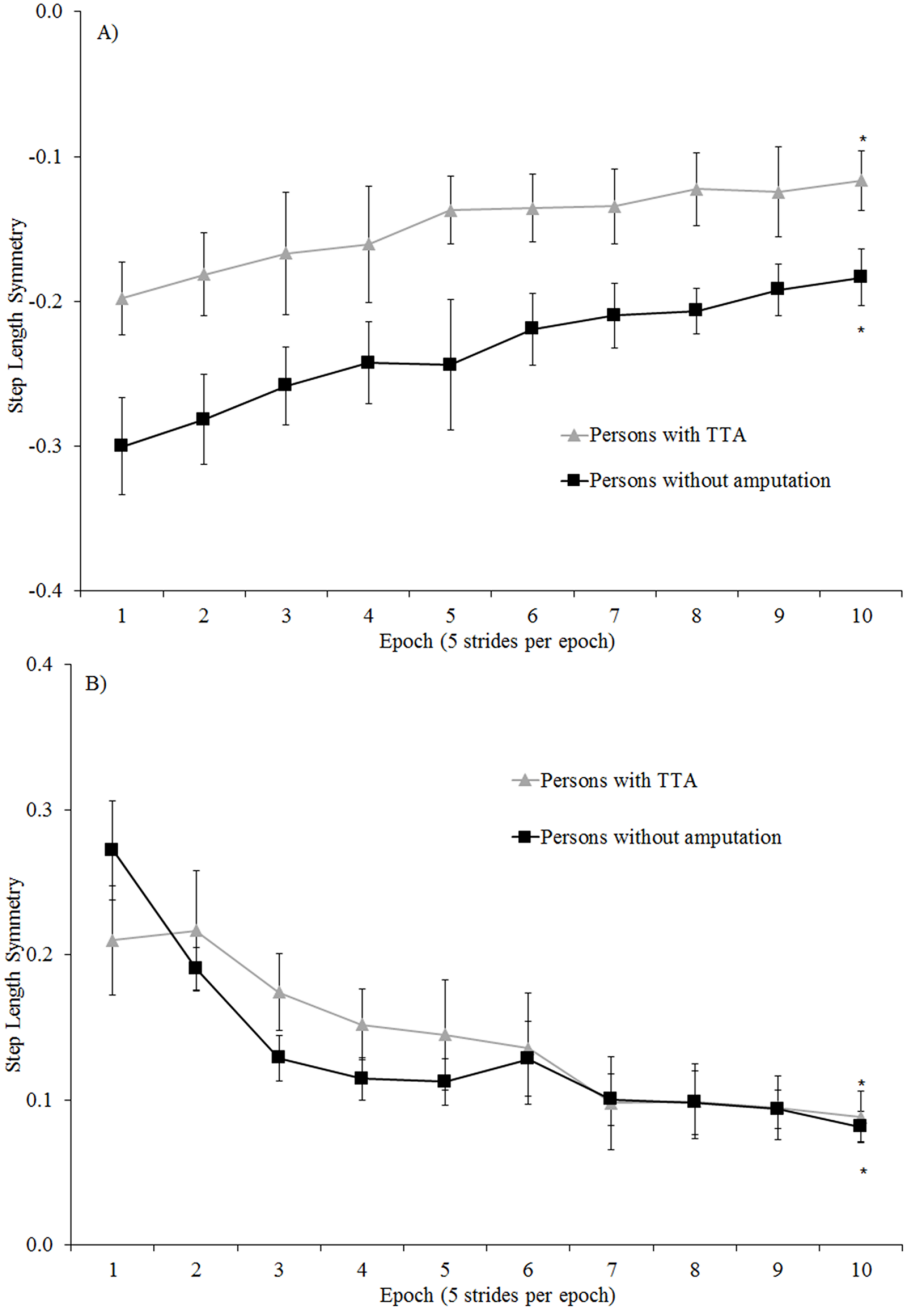
Comparison of step length symmetry across the first 50 strides of: A) split adaptation and B) tied post-adaptation walking conditions for the persons with TTA and persons without an amputation. Error bars indicate standard error. * Indicates within-group differences comparing the 1^st^ and 10^th^ epoch were statistically significant with p <.05. Note: Increased handrail use by persons with TTA resulted in more symmetrical step lengths than the persons without an amputation across the first 50 strides of split adaptation condition.

**Fig 7:**
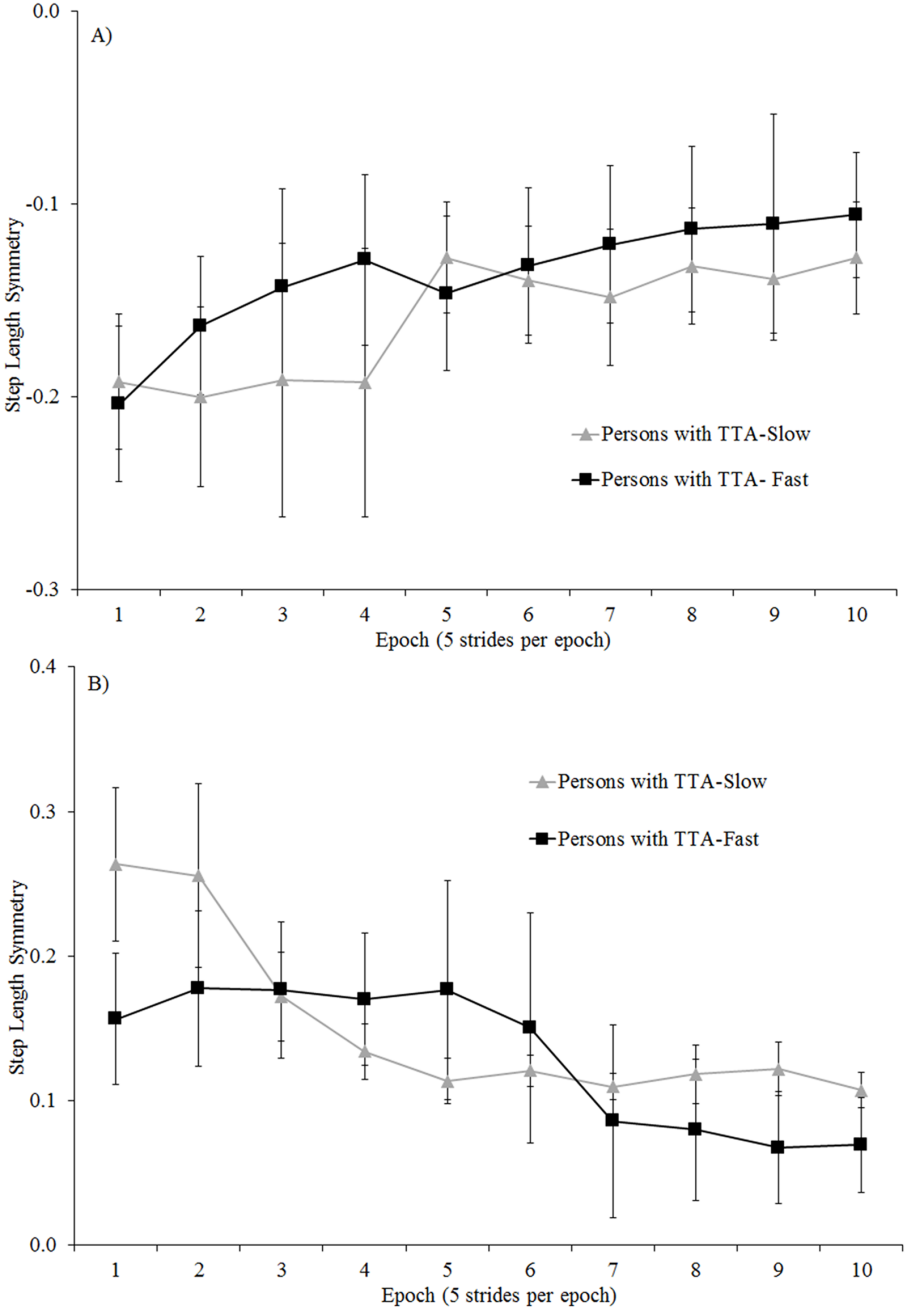
Comparison of step length symmetry across the first 50 strides of: A) split adaptation and B) tied post-adaptation walking conditions for the persons with TTA grouped by belt assignment for the prosthetic limb. Error bars indicate standard error.

All participants significantly improved step length symmetry over the first 50 strides of the split adaptation condition and tied post-adaptation condition (all p <.01; Fig 6). The rates of adaptation and de-adaptation over those strides were similar for persons with and without amputation (non-significant interaction between group and epoch; all p>0.38), and were not different among the persons with TTA based on belt assignment (non-significant interaction between group and epoch; all p>0.30; Fig 7).

## Discussion

Retaining an innate flexibility in motor control strategies for a wide range of walking conditions is an essential aspect of normal locomotor performance. Thus, the primary goal of this study was to evaluate the effects of a unilateral TTA on locomotor adaptability. Results suggest persons with TTA exhibited normal reactive accommodations, and as such were similarly perturbed as those without an amputation. Furthermore, the rates of locomotor adaptation and de-adaptation were similar between persons with and without an amputation, and for the sub-group comparison of persons with TTA based on belt assignment for the prosthetic limb. These findings suggest pathways and processes enabling locomotor accommodation and adaptation either do not depend on unaltered somatosensory feedback from the periphery, or compensations can be made to overcome the altered input.

### Reactive feedback accommodation

At baseline there was little difference in temporal spatial measures between the persons with and without TTA. While the observed highly symmetric walking patterns run counter to the common clinical presentation of persons with TTA[20, 21], the current participants were otherwise healthy and active individuals who underwent extensive physical rehabilitation after a traumatic amputation. Other recent studies with comparable samples have observed locomotor performance similar to persons without an amputation[28, 29]. In all likelihood, the current group of persons with TTA, and those of the prior studies, had a well restored basic walking ability.

Prior research suggested a reactive response in step length symmetry in persons with TTA[18]. The current results provide additional evidence supporting the hypothesis for step length symmetry, as well as being the first to support this hypothesis for limb excursion and stance time symmetry. Our results also showed persons with TTA responded with more symmetric step lengths than persons without an amputation during early split adaptation walking. An ability to maintain greater symmetry during reactive accommodations implies persons with TTA were less perturbed by the onset of split adaptation walking than persons without an amputation. However, persons with TTA may have achieved better symmetry through increased use of the treadmill handrails[30]. Therefore, our interpretation is to suggest the persons with TTA in the current study were likely perturbed to a similar degree by split walking as persons without an amputation. Furthermore, the reactive accommodations were similar regardless of which belt the prosthetic limb was assigned to during split adaptation walking.

Previous research on locomotor adaptability in persons without an amputation offers insights into our finding. Down weighting of sensory input from the lower limb may have allowed the persons with TTA to maintain an accurate internal representation of the body and effectively react to the treadmill belts moving at different speeds[31]. Alternatively, cues from more proximal joints (e.g., the hip) could have either regulated reactive changes, or effectively compensated for the loss of information from the amputated limb, though the former has been questioned[32]. The results also suggest a typical energy storing and return prosthetic foot did not alter the reactive response despite a diminished capacity to control ankle motion and produce net positive power relative to a biological foot-ankle. Overall, these findings suggest alteration of somatosensory input and motor function in the residual limb and prosthetic device do not limit the ability to make reactive accommodations.

### Predictive feedforward adaptation

The feedforward response was primarily evaluated by changes in step length symmetry during split adaptation and tied post-adaptation walking since changes in other parameters (i.e., limb excursion) may not truly reflect “adaptive” processes[13]. Overall, persons with TTA exhibited an adaptive ability and aftereffects in step length symmetry. This result was expected given prior research on locomotor adaptability in persons with TTA[18]. However, the rate of locomotor adaptation/de-adaptation was not reported. Our results indicate persons with TTA demonstrated similar rates of adaptation and de-adaptation in step length symmetry as the group of individuals without amputation. Our results also offer the first evidence the rates of adaptation and de-adaptation are equivalent whether the prosthetic limb is assigned to the slow or fast belt during split-belt walking.

Previous research described the contributions of ankle joint muscles to adaptation for persons without amputation[33, 34]. Nevertheless, limitations of current prosthetic devices were previously found not to impair the adaptability in persons with TTA when the prosthetic limb was assigned to the faster belt[18]. The authors suggested a center of mass (CoM) displacement strategy was employed by the person with TTA to adapt walking. This strategy included allowing the CoM to move further backwards in a global position when on the fast belt of the treadmill, limiting backwards movement when on the slow belt, and regaining forward position during step-to-step transitions from slow belt to fast belt. A lower energy expense to propel the CoM forward when transitioning from a slow moving belt (relative to the energy cost for transitioning from a belt moving at a higher velocity) was cited as a beneficial reason for the CoM displacement strategy. While we did not assess CoM movement in the current study, we did replicate the finding of adaptation in step length symmetry by persons with TTA. Several additional explanations are offered as to why step length symmetry could be adapted while using a prosthetic device, regardless of belt assignment for the prosthetic limb. First, ankle propulsion has a limited effect on locomotor adaptability[33]. As such, the diminished propulsive capacity of energy storing and return feet (relative to a biologic ankle) would not impair adaptability. Second, the primary role of the ankle muscles in adaptation is to set the ankle stiffness in preparation for the predicted perturbation at heel contact[33]. Similar active control of prosthetic stiffness would not be possible. However, it may be speculated the physiological ankle joint stiffness normally created through muscle co-activation was not significantly different than the inherent mechanical stiffness of the prosthetic devices. Third, quadriceps and hamstring muscles demonstrate adaptive changes during split walking[33]. These muscles would be unaffected by a TTA and, possibly, sensorimotor processes at the knee joint could enable the predictive trial and error adjustments in foot placement that improve step length symmetry. Finally, sensory apparatus within the residual limb may, via forces transmitted through the prosthetic device, provide suitable feedback to coordinate adaptive changes through other means.

There was also a potential aftereffect in stance time symmetry for those with TTA that was not observed in those without an amputation. Furthermore, unlike step length symmetry, the response in stance time symmetry was dependent on the belt assignment for split-adaptation walking. Individuals with the prosthetic limb assigned to the slow belt exhibited shorter stance times on the prosthetic limb during tied post-adaptation (0.99±0.02 seconds) than those with the prosthetic assigned to the fast belt (1.23±0.23 seconds). While the exact reason for this difference is unclear, a possible explanation is related to how the nervous system may resolve the difference in sensory input from each limb during split-adaptation walking. Prior research suggests greater importance may be placed on sensory information stemming from the limb on the slow belt since it is in contact with the belt longer than the limb on the fast belt[35]. However, this may not always be the case for persons with TTA. Rather, greater weight may be placed on input from the intact limb or other location due to the limitations in sensory feedback from the amputated limb. As such, generalizing motor patterns to a slow tied post-adaptation walking speed may be lessened when the reference is the intact limb on the fast belt.

### Limitations

We acknowledge the transtibial amputations in our cohort resulted from traumatic injury. Thus, the results may not be fully generalizable to those with amputation at a more proximal anatomical level (ie. transfemoral), or stemming from an etiology (ie. dysvascularity) with greater potential for concomitant systemic neuromuscular impairments. Furthermore, the persons with TTA had all received extensive post amputation rehabilitation and were generally young and very active[36]. We cannot evaluate if adaptability remained intact despite the amputation, or was restored through physical rehabilitation. We also could not full quantify the effect of handrails use on adaptability. While we can confidently suggest the persons with TTA appeared less perturbed during early adaptation because of increased handrail use, we cannot determine how much more perturbed the persons with TTA would have presented absent the handrail use. Finally, small groups (n = 5) were used in evaluating the effect of belt assignment among those with TTA. It is possible some comparisons may have reached statistical significance with data from additional participants. Future research is warranted to address these limitations.

## Conclusions and clinical implications

Persons with TTA exhibit a locomotor adaptability similar to persons without an amputation despite altered somatosensory feedback and functional impairments imposed by use of a prosthetic limb. Therefore, persons with TTA likely have the capacity to modify locomotor strategies to meet the demands of most walking conditions. Our results may also have clinical implications for training interventions aiming to restore locomotor performance in persons with a lower extremity amputation. Split-belt walking has been previously used to correct step length asymmetry[37]. In the current study the persons with TTA exhibited highly symmetric step lengths at baseline so adapting to a more symmetric pattern was not expected. Nevertheless, finding persons with TTA have a normal capacity to adapt locomotor strategies within a single session suggests a multiple session split-belt training program may also be effective for persons with TTA when significant step length asymmetry is present. Moreover, a normal adaptive ability suggests the training volume necessary to alter or acquire a new locomotor strategy is likely unchanged based solely on the presence of a transtibial amputation. In addition, patients may develop greater flexibility and an ability to rapidly change locomotor strategies if interventions that leverage adaptability are used during rehabilitation[10]. Training in such a manner could help maintain stability and safety when faced with challenging walking conditions in everyday life.

## Supporting information

S1 File Supporting data from the individual subjects included in this manuscript. (XLSX)

## References

[1] Munin MC, Espejo-De Guzman MC, Boninger ML, Fitzgerald SG, Penrod LE, Singh J. Predictive factors for successful early prosthetic ambulation among lower-limb amputees. J Rehabil Res Dev. 2001;38(4):379–84. . 11563490

[2] Martin J, Pollock A, Hettinger J. Microprocessor Lower Limb Prosthetics: Review of Current State of the Art. JPO: Journal of Prosthetics and Orthotics. 2010;22(3):183.

[3] Boonstra AM, Schrama J, Fidler V, Eisma WH. The gait of unilateral transfemoral amputees. Scand J Rehabil Med. 1994;26(4):217–23. . 7878397

[4] Sanderson DJ, Martin PE. Lower extremity kinematic and kinetic adaptations in unilateral below-knee amputees during walking. Gait & posture. 1997;6(2):126–36. doi: 10.1016/S0966-6362(97)01112-0

[5] Waters RL, Mulroy S. The energy expenditure of normal and pathologic gait. Gait & posture. 1999;9(3):207–31. . 10575082 10.1016/s0966-6362(99)00009-0

[6] Genin JJ, Bastien GJ, Franck B, Detrembleur C, Willems PA. Effect of speed on the energy cost of walking in unilateral traumatic lower limb amputees. Eur J Appl Physiol. 2008;103(6):655–63. doi: 10.1007/s00421-008-0764-0 . 18478251 10.1007/s00421-008-0764-0

[7] Grillner S, Wallen P. Central pattern generators for locomotion, with special reference to vertebrates. Annu Rev Neurosci. 1985;8:233–61. doi: 10.1146/annurev.ne.08.030185.001313 . 2984978 10.1146/annurev.ne.08.030185.001313

[8] Dietz V. Proprioception and locomotor disorders. Nature reviews Neuroscience. 2002;3(10):781–90. Epub 2002/10/03. doi: 10.1038/nrn939 . 12360322 10.1038/nrn939

[9] Morton SM, Bastian AJ. Cerebellar contributions to locomotor adaptations during splitbelt treadmill walking. J Neurosci. 2006;26(36):9107–16. doi: 10.1523/JNEUROSCI.2622-06.2006 . 16957067 10.1523/JNEUROSCI.2622-06.2006 PMC6674518

[10] Reisman DS, Bastian AJ, Morton SM. Neurophysiologic and rehabilitation insights from the split-belt and other locomotor adaptation paradigms. Physical therapy. 2010;90(2):187–95. Epub 2009/12/22. doi: 10.2522/ptj.20090073 . 20023001 10.2522/ptj.20090073 PMC2816031

[11] Reisman DS, Block HJ, Bastian AJ. Interlimb coordination during locomotion: what can be adapted and stored? J Neurophysiol. 2005;94(4):2403–15. Epub 2005/06/17. doi: 10.1152/jn.00089.2005 . 15958603 10.1152/jn.00089.2005

[12] Roemmich RT, Nocera JR, Stegemoller EL, Hassan A, Okun MS, Hass CJ. Locomotor adaptation and locomotor adaptive learning in Parkinson's disease and normal aging. Clin Neurophysiol. 2014;125(2):313–9. doi: 10.1016/j.clinph.2013.07.003 . 23916406 10.1016/j.clinph.2013.07.003 PMC3844121

[13] Bruijn SM, Van Impe A, Duysens J, Swinnen SP. Split-belt walking: adaptation differences between young and older adults. J Neurophysiol. 2012;108(4):1149–57. Epub 2012/05/25. doi: 10.1152/jn.00018.2012 . 22623488 10.1152/jn.00018.2012 PMC3424083

[14] Reisman DS, Wityk R, Silver K, Bastian AJ. Locomotor adaptation on a split-belt treadmill can improve walking symmetry post-stroke. Brain. 2007;130(Pt 7):1861–72. Epub 2007/04/05. doi: 10.1093/brain/awm035 . 17405765 10.1093/brain/awm035 PMC2977955

[15] Vasudevan EV, Glass RN, Packel AT. Effects of traumatic brain injury on locomotor adaptation. Journal of neurologic physical therapy: JNPT. 2014;38(3):172–82. doi: 10.1097/NPT.0000000000000049 . 24892766 10.1097/NPT.0000000000000049

[16] Mueller MJ, Salsich GB, Bastian AJ. Differences in the gait characteristics of people with diabetes and transmetatarsal amputation compared with age-matched controls. Gait & posture. 1998;7(3):200–6. Epub 1999/04/14. . 10200385 10.1016/s0966-6362(98)00015-0

[17] Mohammadi F, Bruijn SM, Vervoort G, van Wegen EE, Kwakkel G, Verschueren S, et al Motor switching and motor adaptation deficits contribute to freezing of gait in Parkinson's disease. Neurorehabil Neural Repair. 2015;29(2):132–42. doi: 10.1177/1545968314545175 . 25416741 10.1177/1545968314545175

[18] Selgrade BP, Toney ME, Chang YH. Two biomechanical strategies for locomotor adaptation to split-belt treadmill walking in subjects with and without transtibial amputation. J Biomech. 2017;53:136–43. doi: 10.1016/j.jbiomech.2017.01.012 . 28126335 10.1016/j.jbiomech.2017.01.012 PMC5340589

[19] Kannape OA, Herr HM. Split-belt adaptation and gait symmetry in transtibial amputees walking with a hybrid EMG controlled ankle-foot prosthesis. Conference proceedings: Annual International Conference of the IEEE Engineering in Medicine and Biology Society IEEE Engineering in Medicine and Biology Society Conference. 2016;2016:5469–72. doi: 10.1109/EMBC.2016.7591964 . 28269495 10.1109/EMBC.2016.7591964

[20] Isakov E, Burger H, Krajnik J, Gregoric M, Marincek C. Double-limb support and step-length asymmetry in below-knee amputees. Scand J Rehabil Med. 1997;29(2):75–9. . 9198256

[21] Hak L, van Dieen JH, van der Wurff P, Houdijk H. Stepping asymmetry among individuals with unilateral transtibial limb loss might be functional in terms of gait stability. Physical therapy. 2014;94(10):1480–8. doi: 10.2522/ptj.20130431 . 24903115 10.2522/ptj.20130431

[22] Malone LA, Vasudevan EV, Bastian AJ. Motor adaptation training for faster relearning. J Neurosci. 2011;31(42):15136–43. doi: 10.1523/JNEUROSCI.1367-11.2011 . 22016547 10.1523/JNEUROSCI.1367-11.2011 PMC3209529

[23] Malone LA, Bastian AJ. Thinking about walking: effects of conscious correction versus distraction on locomotor adaptation. J Neurophysiol. 2010;103(4):1954–62. Epub 2010/02/12. doi: 10.1152/jn.00832.2009 . 20147417 10.1152/jn.00832.2009 PMC2853281

[24] Torres-Oviedo G, Bastian AJ. Seeing is believing: effects of visual contextual cues on learning and transfer of locomotor adaptation. J Neurosci. 2010;30(50):17015–22. Epub 2010/12/17. doi: 10.1523/JNEUROSCI.4205-10.2010 . 21159971 10.1523/JNEUROSCI.4205-10.2010 PMC3025449

[25] Roemmich RT, Long AW, Bastian AJ. Seeing the Errors You Feel Enhances Locomotor Performance but Not Learning. Curr Biol. 2016;26(20):2707–16. doi: 10.1016/j.cub.2016.08.012 . 27666970 10.1016/j.cub.2016.08.012 PMC5081226

[26] Wilken JM, Rodriguez KM, Brawner M, Darter BJ. Reliability and Minimal Detectible Change values for gait kinematics and kinetics in healthy adults. Gait & posture. 2012;35(2):301–7. Epub 2011/11/02. doi: 10.1016/j.gaitpost.2011.09.105 . 22041096 10.1016/j.gaitpost.2011.09.105

[27] Malone LA, Bastian AJ. Age-related forgetting in locomotor adaptation. Neurobiol Learn Mem. 2016;128:1–6. doi: 10.1016/j.nlm.2015.11.003 . 26589520 10.1016/j.nlm.2015.11.003 PMC4839585

[28] Esposito ER, Rodriguez KM, Rabago CA, Wilken JM. Does unilateral transtibial amputation lead to greater metabolic demand during walking? J Rehabil Res Dev. 2014;51(8):1287–96. doi: 10.1682/JRRD.2014.06.0141 . 25671680 10.1682/JRRD.2014.06.0141

[29] Gates DH, Scott SJ, Wilken JM, Dingwell JB. Frontal plane dynamic margins of stability in individuals with and without transtibial amputation walking on a loose rock surface. Gait & posture. 2013;38(4):570–5. Epub 2013/03/14. doi: 10.1016/j.gaitpost.2013.01.024 . 23481866 10.1016/j.gaitpost.2013.01.024 PMC3720773

[30] T IJ, Lamoth CJ, Houdijk H, Tolsma M, van der Woude LH, Daffertshofer A, et al Effects of handrail hold and light touch on energetics, step parameters, and neuromuscular activity during walking after stroke. Journal of neuroengineering and rehabilitation. 2015;12:70 doi: 10.1186/s12984-015-0051-3 . 26298647 10.1186/s12984-015-0051-3 PMC4546819

[31] Layne CS, Chelette AM, Pourmoghaddam A. Impact of altered lower limb proprioception produced by tendon vibration on adaptation to split-belt treadmill walking. Somatosens Mot Res. 2015;32(1):31–8. doi: 10.3109/08990220.2014.949007 . 25162146 10.3109/08990220.2014.949007

[32] Hoogkamer W, Bruijn SM, Potocanac Z, Van Calenbergh F, Swinnen SP, Duysens J. Gait asymmetry during early split-belt walking is related to perception of belt speed difference. J Neurophysiol. 2015;114(3):1705–12. doi: 10.1152/jn.00937.2014 . 26203114 10.1152/jn.00937.2014 PMC4567612

[33] Ogawa T, Kawashima N, Ogata T, Nakazawa K. Predictive control of ankle stiffness at heel contact is a key element of locomotor adaptation during split-belt treadmill walking in humans. J Neurophysiol. 2014;111(4):722–32. doi: 10.1152/jn.00497.2012 . 24225544 10.1152/jn.00497.2012

[34] Finley JM, Bastian AJ, Gottschall JS. Learning to be economical: the energy cost of walking tracks motor adaptation. The Journal of physiology. 2013;591(4):1081–95. Epub 2012/12/19. doi: 10.1113/jphysiol.2012.245506 . 23247109 10.1113/jphysiol.2012.245506 PMC3591716

[35] Vasudevan EV, Bastian AJ. Split-belt treadmill adaptation shows different functional networks for fast and slow human walking. J Neurophysiol. 2010;103(1):183–91. doi: 10.1152/jn.00501.2009 . 19889853 10.1152/jn.00501.2009 PMC2807217

[36] Gajewski D, Granville R. The United States Armed Forces Amputee Patient Care Program. J Am Acad Orthop Surg. 2006;14(10 Spec No.):S183–7. Epub 2006/09/28. . 17003196 10.5435/00124635-200600001-00040

[37] Reisman DS, McLean H, Keller J, Danks KA, Bastian AJ. Repeated split-belt treadmill training improves poststroke step length asymmetry. Neurorehabil Neural Repair. 2013;27(5):460–8. Epub 2013/02/09. doi: 10.1177/1545968312474118 . 23392918 10.1177/1545968312474118 PMC3738184

